# CT-derived 3D-diaphragm motion in emphysema and IPF compared to normal subjects

**DOI:** 10.1038/s41598-021-93980-5

**Published:** 2021-07-21

**Authors:** Ji Hee Kang, Jiwoong Choi, Kum Ju Chae, Kyung Min Shin, Chang-Hoon Lee, Junfeng Guo, Ching-Long Lin, Eric A. Hoffman, Changhyun Lee

**Affiliations:** 1 Department of Radiology, Konkuk University Medical Center, Seoul, Korea; 2 Department of Internal Medicine, School of Medicine, University of Kansas, 3901 Rainbow Blvd, Kansas City, KS 66160 USA; 3 Department of Bioengineering, University of Kansas, Lawrence, KS USA; 4 Department of Radiology, Jeonbuk National University Hospital, Jeonju, Korea; 5 Department of Radiology, Kyungpook National University, Daegu, Korea; 6 Division of Pulmonary and Critical Care Medicine, Department of Internal Medicine, Seoul National University Hospital, Seoul, South Korea; 7 Department of Radiology, University of Iowa, Iowa City, IA USA; 8 Roy J. Carver Department of Biomedical Engineering, University of Iowa, Iowa City, IA USA; 9 Department of Mechanical Engineering, IIHR-Hydroscience and Engineering, University of Iowa, Iowa City, IA USA; 10 Department of Medicine, University of Iowa, Iowa City, IA USA; 11 Department of Radiology, Seoul National University College of Medicine, 101 Daehangno, Jongno-gu, Seoul, 03080 Korea

**Keywords:** Computational biophysics, Biological physics, Respiratory tract diseases

## Abstract

Image registration-based local displacement analysis enables evaluation of respiratory motion between two computed tomography-captured lung volumes. The objective of this study was to compare diaphragm movement among emphysema, idiopathic pulmonary fibrosis (IPF) and normal subjects. 29 normal, 50 emphysema, and 51 IPF subjects were included. A mass preserving image registration technique was used to compute displacement vectors of local lung regions at an acinar scale. Movement of the diaphragm was assumed to be equivalent to movement of the basal lung within 5 mm from the diaphragm. Magnitudes and directions of displacement vectors were compared between the groups. Three-dimensional (3D) and apico-basal displacements were smaller in emphysema than normal subjects (*P* = 0.003, *P* = 0.002). Low lung attenuation area on expiration scan showed significant correlations with decreased 3D and apico-basal displacements (r = − 0.546, *P* < 0.0001; r = − 0.521, *P* < 0.0001) in emphysema patients. Dorsal–ventral displacement was smaller in IPF than normal subjects (*P* < 0.0001). The standard deviation of the displacement angle was greater in both emphysema and IPF patients than normal subjects (*P* < 0.0001). In conclusion, apico-basal movement of the diaphragm is reduced in emphysema while dorsal–ventral movement is reduced in IPF. Image registration technique to multi-volume CT scans provides insight into the pathophysiology of limited diaphragmatic motion in emphysema and IPF.

## Introduction

The diaphragm is a respiratory muscle which accounts for approximately 80% of all respiratory work in normal tidal breathing^1^. Diaphragmatic dysfunction has been shown to be frequently associated with lung diseases such as emphysema or idiopathic pulmonary fibrosis (IPF)^2–5^. Various factors including oxidative stress, sarcomeric injury, hypoxia, and systemic inflammation attribute to diaphragm weakness in those lung diseases^6–8^. The weakening of the diaphragm in patients with respiratory disease is clinically significant because respiratory failure due to diaphragm dysfunction is associated with increased mortality and worse prognosis^9,10^.

To analyze diaphragmatic mobility in patients with respiratory diseases such as emphysema or IPF, previous studies utilized magnetic resonance imaging (MRI)^3,11^, ultrasound^4^, dynamic chest radiography^12^, or fluoroscopy^13^ to measure two-dimensional (2D) diaphragmatic excursions. However, the actual respiratory motions are three-dimensional (3D). In 2009, Yin et al.^14^ developed a mass preserving non-rigid image registration methods to obtain local-to-local matching of two computed tomography (CT) acquired at different inspiration levels, using the sum of squared tissue volume difference (SSTVD) approach. This image registration method provides a robust map of local lung displacement vectors, which enables evaluation of 3D respiratory motions during large deformation between inspiratory and expiratory CTs. This quantitative CT (QCT) approach successfully differentiated deformation characteristics between asthmatic and healthy human lungs^15^. To our knowledge, 3D respiratory motions of emphysema or IPF have not been elucidated yet.

Therefore, the purpose of this study is to compare diaphragm movement among emphysema, IPF and normal subjects using image registration based local displacement technique, “lung motionography”.

## Results

### Displacement vectors

**Figure 1:**
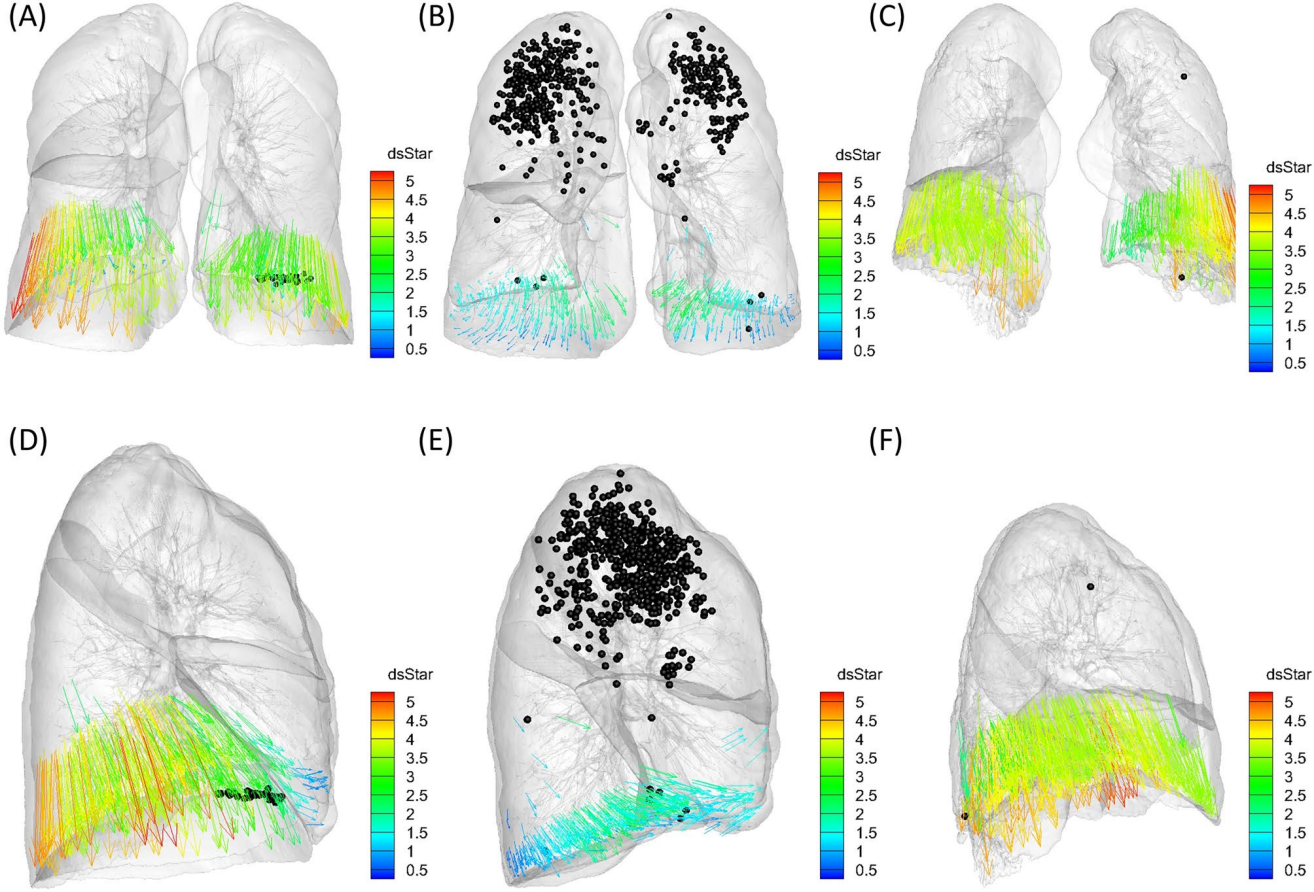
Demonstrative images of the three subject groups from anteroposterior and lateral views. Arrows are displacement vectors of the diaphragm which are color-coded by their magnitude (‘dsStar’ written on the right represents the magnitude of 3D displacement vector). Black spheres represent LAA_insp_ (area of the lungs in which attenuation is less than -950 Hounsfield units [HU] on inspiration scan). (**A**,**D**) Normal. (**B**,**E**) Emphysema. The magnitude of 3D and apico-basal displacements is smaller than that of the normal subject. Note the diaphragm flattening on the lateral view. (**C**,**F**) IPF. Dorsal–ventral displacement is decreased.

**Figure 2:**
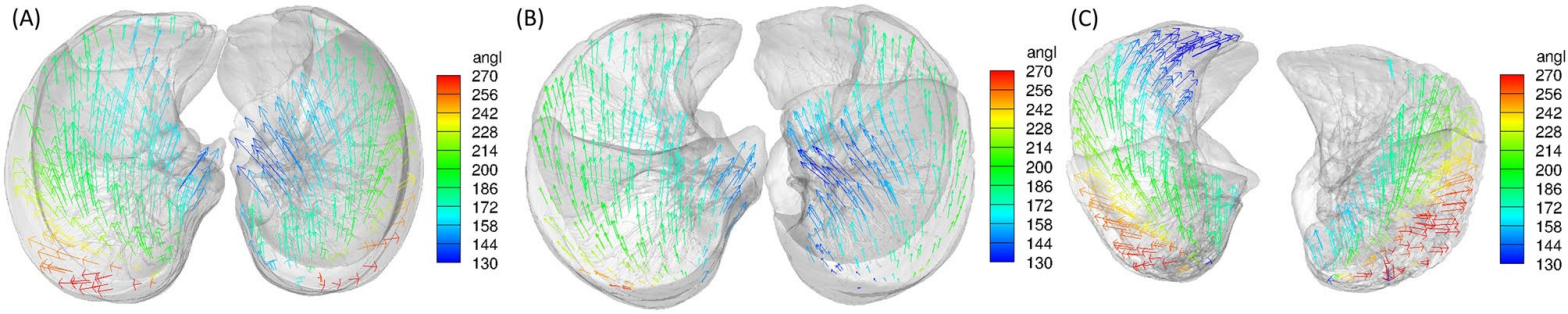
Demonstrative images of the three subject groups viewed from the below. Arrows are displacement vectors of the diaphragm which are color-coded by the displacement angle (‘angl’ written on the right represents the angle of dorsoventral-transverse displacement vector). (**A**) Normal. (**B**) Emphysema. The posteromedial portion of the diaphragm shows smaller displacement angle than normal controls. (**C**) IPF. The anteromedial portion of the diaphragm shows smaller displacement angle than normal controls. Note that heterogeneous displacement angles are demonstrated especially in the posterior portion of the left diaphragm in this patient.

Table 1 demonstrates the results of the comparison among subject groups (Figs. 1, 2). In general, emphysema patients showed smaller 3D and apico-basal displacements. Meanwhile, IPF patients had a tendency of smaller dorsal–ventral displacement than other groups.

**Table 1:** Results of the comparison among normal, emphysema, and IPF groups.

Displacement	Normal (n = 29)	Emphysema (n = 50)	IPF (n = 51)	ANOVA *P* value	Post-hoc comparison *P* value		
					Normal-Emphysema	Normal-IPF	Emphysema-IPF
**Whole diaphragm**							
3D	3.50	2.79 (0.003)*	2.72 (0.019)*	0.035*	0.054	0.043*	0.783
Transverse	0.12	0.002 (0.006)*	0.09 (0.539)	0.083	N/A	N/A	N/A
Apico-basal	3.17	2.40 (0.002)*	2.47 (0.026)*	0.027*	0.033*	0.042*	0.764
Dorsal–ventral	1.17	0.99 (0.052)	0.71 (< 0.0001)*	< 0.0001*	0.065	< 0.0001*	0.001*
Angle (mean, degree)	189.54	186.72 (0.215)	192.09 (0.422)	0.195	N/A	N/A	N/A
Angle (SD)	27.28	37.51 (< 0.0001)*	48.31 (< 0.0001)*	< 0.0001*	0.008*	< 0.0001*	0.002*
Diaphragm thickness (mm)	4.52	3.95 (0.030)*	3.97 (0.043)*	0.068	N/A	N/A	N/A
**Quadrant 1 (Antero-lateral)**							
3D	3.30	2.52 (0.002)*	2.64 (0.055)	0.029*	0.031*	0.057	0.640
Transverse	0.08	0.07 (0.835)	0.09 (0.847)	0.901	N/A	N/A	N/A
Apico-basal	2.88	2.06 (0.002)*	2.31 (0.070)	0.020*	0.016*	0.098	0.320
Dorsal–ventral	1.40	1.06 (0.006)*	1.05 (0.021)*	0.023*	0.035*	0.035*	0.941
Angle (mean, degree)	184.82	187.16 (0.468)	188.36 (0.400)	0.775	N/A	N/A	N/A
Angle (SD)	18.04	26.77 (0.003)*	27.45 (0.003)*	0.037*	0.052	0.049*	0.836
**Quadrant 2 (Antero-medial)**							
3D	2.67	2.27 (0.072)	2.19 (0.108)	0.172	N/A	N/A	N/A
Transverse	0.30	0.29 (0.930)	0.41 (0.299)	0.406	N/A	N/A	N/A
Apico-basal	2.19	1.57 (0.006)*	1.85 (0.220)	0.043*	0.039*	0.344	0.344
Dorsal–ventral	1.24	1.29 (0.698)	0.71 (< 0.0001)*	< 0.0001*	0.704	< 0.0001*	< 0.0001*
Angle (mean, degree)	172.33	173.66 (0.677)	156.85 (0.011)*	0.002*	0.825	0.021*	0.004*
Angle (SD)	20.13	24.85 (0.175)	24.97 (0.237)	0.547	N/A	N/A	N/A
**Quadrant 3 (Postero-medial)**							
3D	3.53	2.91 (0.015)*	2.75 (0.030)*	0.080	N/A	N/A	N/A
Transverse	0.14	0.30 (0.004)*	0.14 (0.988)	0.079	N/A	N/A	N/A
Apico-basal	3.29	2.57 (0.005)*	2.57 (0.032)*	0.054	N/A	N/A	N/A
Dorsal–ventral	1.08	1.08 (0.983)	0.60 (< 0.0001)*	< 0.0001*	0.983	< 0.0001*	< 0.0001*
Angle (mean, degree)	177.24	168.60 (0.005)*	181.55 (0.335)	0.004*	0.121	0.344	0.003*
Angle (SD)	22.19	25.34 (0.205)	47.82 (< 0.0001)*	< 0.0001*	0.432	< 0.0001*	< 0.0001*
**Quadrant 4 (Postero-lateral)**							
3D	3.94	3.19 (0.004)*	2.97 (0.009)*	0.024*	0.073	0.022*	0.476
Transverse	0.55	0.32 (< 0.0001)*	0.46 (0.246)	0.003*	0.004*	0.195	0.039*
Apico-basal	3.70	2.99 (0.009)*	2.78 (0.011)*	0.030*	0.088	0.027*	0.478
Dorsal–ventral	1.01	0.69 (< 0.0001)*	0.52 (< 0.0001)*	< 0.0001*	0.001*	< 0.0001*	0.035
Angle (mean, degree)	210.69	206.76 (0.262)	216.99 (0.154)	0.044*	0.412	0.376	0.040*
Angle (SD)	19.66	40.28 (< 0.0001)*	38.73 (< 0.0001)*	< 0.0001*	< 0.0001*	< 0.0001*	0.715

Numbers in the parentheses are *P* values from the independent t-test. For post-hoc analysis, *P* values were adjusted by holm method when the ANOVA test was statistically significant. *P* values with asterisk* indicate to be statistically significant

N/A = not applicable.

For displacement angles of the whole diaphragm, there was no significant difference between both patient groups and normal controls (189.54 degrees in normal versus 186.72 degrees in emphysema [*P* = 0.215], and 192.09 degrees in IPF [*P* = 0.422]). However, the posteromedial quadrant in emphysema patients and the anteromedial quadrant in IPF subjects demonstrated significantly smaller displacement angle than normal controls (168.60 versus 177.24 degrees, *P* = 0.005; 156.85 versus 172.33 degrees, *P* = 0.011). The standard deviation (SD) of displacement angles in both emphysema and IPF groups was greater compared to normal subjects.

### Diaphragm thickness

Diaphragm thicknesses of both emphysema and IPF patients were smaller than that of the normal subjects (3.95 mm versus 4.52 mm, *P* = 0.030; 3.97 mm versus 4.52 mm, *P* = 0.043). There were no significant differences between emphysema and IPF patients. However, analysis of variance (ANOVA) analysis found no difference in diaphragm thickness among the three groups.

### Emphysema distribution and diaphragm displacement

Results of univariate analyses between upper lobe predominant and diffuse emphysema types are summarized in Table 2. 3D and apico-basal displacements were smaller in the diffuse type than upper lobe predominant type without statistical significance (2.72 versus 2.88, *P* = 0.384; 2.33 versus 2.47, *P* = 0.460).

**Table 2:** Results of univariate analysis between upper lobe predominant and diffuse type emphysema.

Displacement	Upper lobe predominant	Diffuse	*P* Value
3D	2.88	2.72	0.384
Transverse	0.05	0.06	0.059
Apico-basal	2.47	2.33	0.460
Dorsal–ventral	1.04	0.98	0.505
Angle (mean, degree)	187.97	184.85	0.356
Angle (SD)	32.80	35.85	0.305

### Correlation of disease severity and displacement vectors

**Figure 3:**
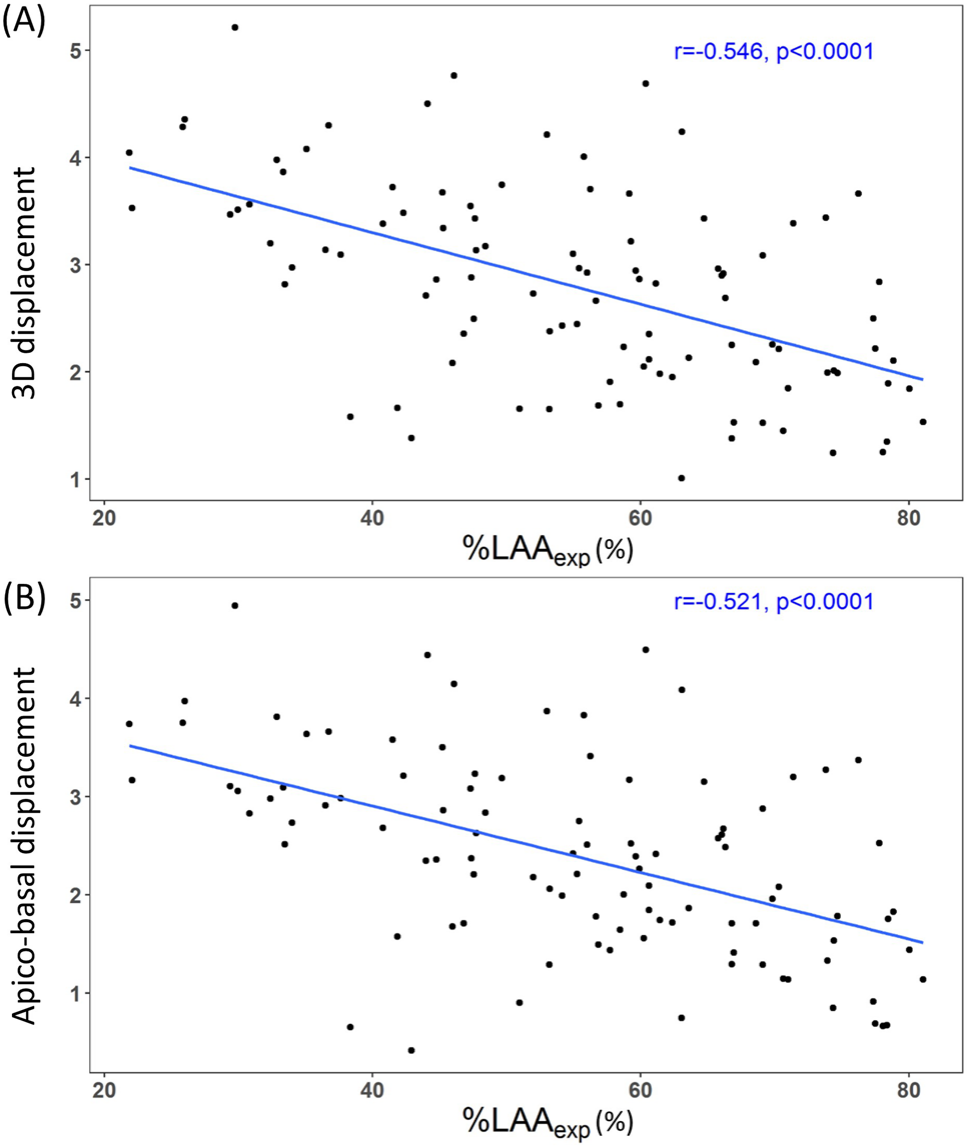
Correlation between %LAA_exp_ and magnitude of displacement vectors in emphysema patients. %LAA_exp_ (%) represents percentage of lung volume on expiration scan with attenuation less than -856 Hounsfield units (HU). (**A**) Correlation between %LAA_exp_ (%) and 3D displacement of the diaphragm. %LAA_exp_ (%) is negatively correlated with 3D displacement of the diaphragm (r = − 0.546, *P* < 0.0001). (**B**) Correlation between %LAA_exp_ (%) and apico-basal displacement of the diaphragm. %LAA_exp_ (%) is negatively correlated with apico-basal displacement of the diaphragm (r = − 0.521, *P* < 0.0001).

**Figure 4:**
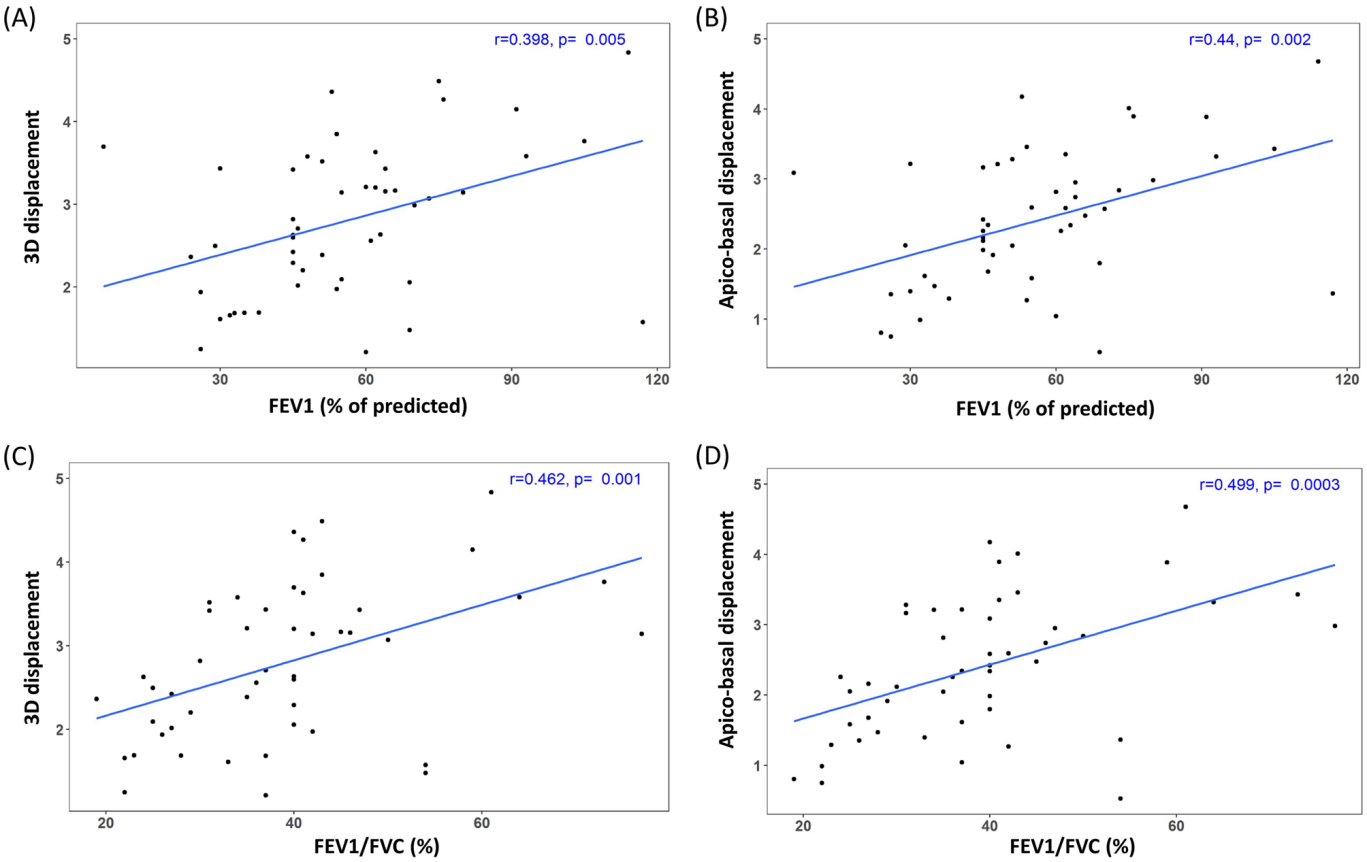
Correlation between pulmonary function test variables and magnitude of displacement vectors in emphysema patients. (**A**,**B**) Correlation between forced expiratory volume in 1 s (FEV1 [% of predicted]) and displacement of the diaphragm. (**A**) FEV1 (% of predicted) is positively correlated with 3D displacement of the diaphragm (r = 0.398, *P* = 0.005). (**B**) FEV1 (% of predicted) is positively correlated with apico-basal displacement of the diaphragm (r = 0.44, *P* = 0.002). (**C**,**D**) Correlation between FEV1 / forced vital capacity (FVC) (%) and displacement of the diaphragm. (**C**) FEV1/FVC (%) is positively correlated with 3D displacement of the diaphragm (r = 0.462, *P* = 0.001). (**D**) FEV1/FVC (%) is positively correlated with apico-basal displacement of the diaphragm (r = 0.499, *P* = 0.0003).

Table 3 shows the results of correlation test between low attenuation area (LAA) and parameters of displacement vectors in emphysema patients. Lung volume percentage of the LAA on expiration scan (%LAA_exp_, percentage of lung volume on expiration scan with attenuation less than −856 Hounsfield units [HU]) showed negative correlation with 3D, apico-basal, and dorsal–ventral displacement (*P* < 0.0001, r = − 0.546; *P* < 0.0001, r = − 0.521; *P* < 0.0001, r = − 0.387) (Fig. 3). Lung volume percentage of the LAA on inspiration scan (%LAA_insp_, percentage of lung volume at total lung capacity [TLC] with attenuation less than -950HU) had significant negative correlation with 3D and apico-basal displacement (*P* = 0.020, r = − 0.233; *P* = 0.015, r = − 0.242). Among pulmonary function test (PFT) parameters, forced expiratory volume (FEV) in 1 s (FEV1) and FEV1/forced vital capacity (FVC) demonstrated a significant positive correlation with 3D and apico-basal displacement (Fig. 4).

**Table 3:** Results of correlation test between %LAA and magnitude, angle of displacement vectors in emphysema patients.

Displacement	%LAA_insp_		%LAA_exp_	
	Correlation coefficient (r)	*P* Value	Correlation coefficient (r)	*P* Value
3D	− 0.233	0.020*	− 0.546	< 0.0001*
Transverse	− 0.041	0.684	− 0.045	0.657
Apico-basal	− 0.242	0.015*	− 0.521	< 0.0001*
Dorsal–ventral	− 0.142	0.158	− 0.387	< 0.0001*
Angle (mean)	− 0.107	0.291	− 0.121	0.230
Angle (SD)	− 0.083	0.409	− 0.020	0.840

*P* values with asterisk* indicate to be statistically significant.

**Figure 5:**
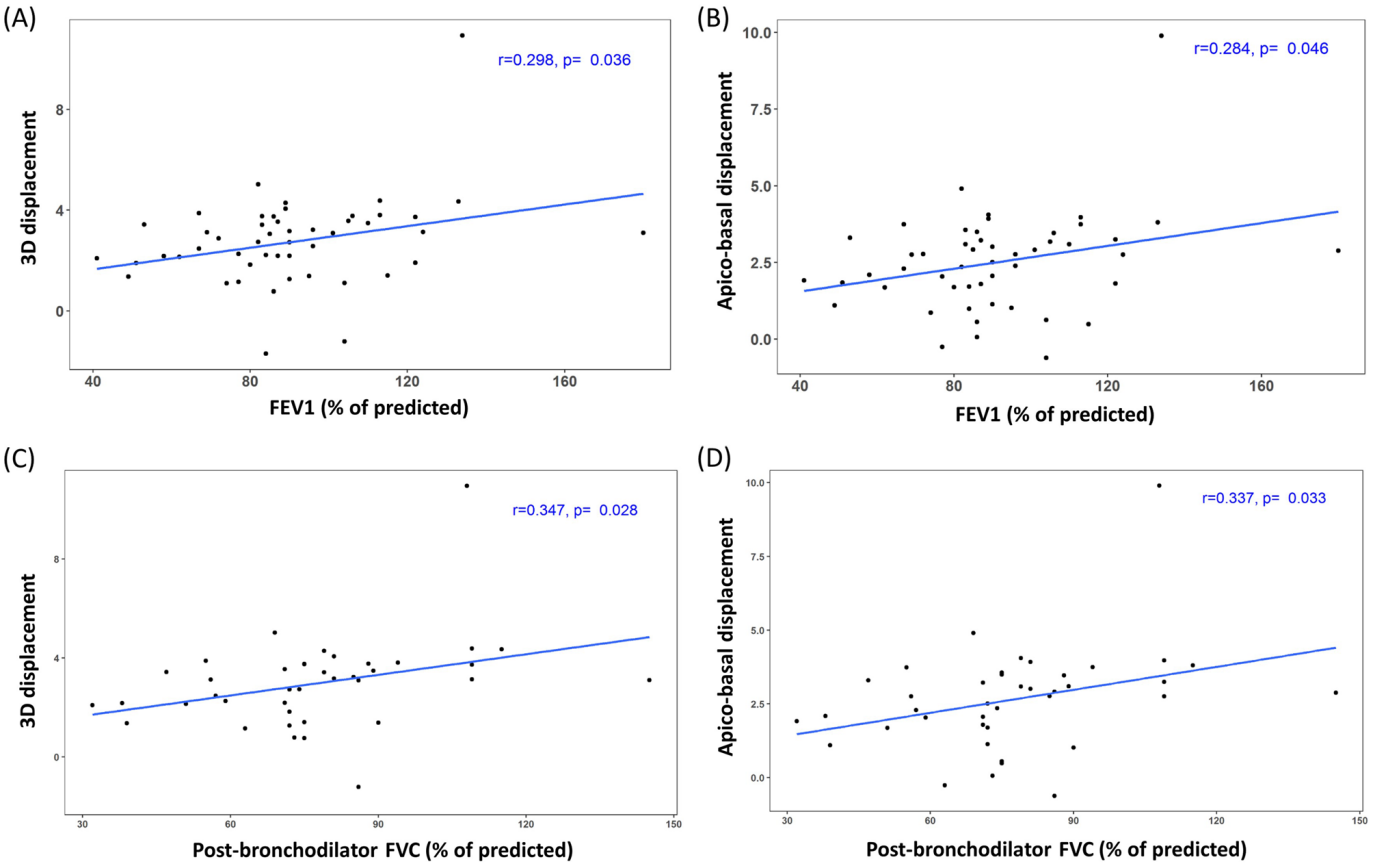
Correlation between pulmonary function test variables and magnitude of displacement vectors in IPF patients. (**A**,**B**) Correlation between forced expiratory volume in 1 s (FEV1 [% of predicted]) and displacement of the diaphragm. (**A**) FEV1 (% of predicted) is positively correlated with 3D displacement of the diaphragm (r = 0.298, *P* = 0.036). (**B**) FEV1 (% of predicted) is positively correlated with apico-basal displacement of the diaphragm (r = 0.284, *P* = 0.046). (**C**,**D**) Correlation between forced vital capacity after the administration of bronchodilator (Post-bronchodilator FVC) and displacement of the diaphragm. (**C**) Post-bronchodilator FVC (% of predicted) is positively correlated with 3D displacement of the diaphragm (r = 0.347, *P* = 0.028). (**D**) Post-bronchodilator FVC (% of predicted) is positively correlated with apico-basal displacement of the diaphragm (r = 0.337, *P* = 0.033).

Table 4 demonstrates the results of correlation tests between IPF scores and parameters of the displacement vectors. Visual ground glass opacities (GGO) score had a negative correlation with 3D and apico-basal displacement (*P* = 0.032, r = − 0.213; *P* = 0.014, r = − 0.244). The sum of reticular and honeycombing scores and the total score objectively measured by the Adaptive Multiple Features Method (AMFM) software demonstrated a negative correlation with apico-basal displacement (*P* = 0.049, r = − 0.208; *P* = 0.032, r = − 0.227). FEV1 and post-bronchodilator FVC showed significant positive correlation with 3D and apico-basal displacement (Fig. 5).

**Table 4:** Correlation coefficient (r) between IPF score and magnitude, angle of displacement vectors.

Displacement	Reticular + Honeycombing		GGO		Total score	
	Visual	AMFM	Visual	AMFM	Visual	AMFM
3D	− 0.101 (0.314)	− 0.157 (0.138)	− 0.213 (0.032)*	− 0.115 (0.282)	− 0.142 (0.156)	− 0.160 (0.132)
Transverse	0.024 (0.813)	− 0.040 (0.705)	0.014 (0.885)	− 0.116 (0.277)	0.025 (0.803)	− 0.075 (0.484)
Apico-basal	− 0.114 (0.255)	− 0.208 (0.049)*	− 0.244 (0.014)*	− 0.190 (0.073)	− 0.161 (0.107)	− 0.227 (0.032)*
Dorsal–ventral	− 0.085 (0.398)	− 0.198 (0.062)	− 0.098 (0.329)	− 0.151 (0.156)	− 0.100 (0.319)	− 0.204 (0.054)
Angle (mean)	− 0.028 (0.780)	− 0.108 (0.310)	− 0.027 (0.787)	− 0.109 (0.308)	− 0.032 (0.751)	− 0.122 (0.254)
Angle (SD)	− 0.056 (0.577)	− 0.033 (0.754)	− 0.056 (0.576)	− 0.089 (0.407)	− 0.064 (0.523)	− 0.059 (0.581)

Numbers in parenthesis are *P* values. *P* values with asterisk* indicate to be statistically significant.

*AMFM* adaptive multiple features method.

## Discussion

Chest CT is the imaging modality of choice for assessing various pulmonary diseases. It is a non-invasive imaging technique, which provides excellent anatomical details of the lungs and airways. Recently, a variety of research has been conducted to quantify visual CT features. By registering two CT images obtained at different lung volume, automated and quantified CT analysis is feasible. Nishio et al.^16^ reported that airflow limitation in smokers could be accurately evaluated by air trapping images generated from paired inspiratory and expiratory CT images. Galban et al.^17^ and Ho et al.^18^ adopted parametric response mapping as a CT-based biomarker to diagnose specific COPD phenotypes or to develop a deep learning model diagnosing COPD. In addition, registration of two CT images from two time points provides temporal subtraction images to detect changes in pulmonary nodules^19^. In this study, we demonstrated alteration of diaphragm movement in emphysema and IPF, and its correlation with disease severity.

In emphysema patients, weakened diaphragmatic contractility due to oxidative stress or systemic inflammation might attribute to the decreased diaphragmatic motion. We speculate that air-trapping of the lung is the major cause of the diaphragmatic movement reduction, especially in the apico-basal direction. As demonstrated in our study, normal diaphragm motion was greater in the apico-basal direction (3.17) as compared with any other directions (dorsal–ventral direction = 1.17, transverse direction = 0.12). Therefore, we assumed that if inhaled air cannot be emitted in the expiratory phase, apico-basal movement would be most affected. Consistent with our hypothesis, our study demonstrated that decreased diaphragmatic motions in 3D and in the apico-basal direction were significantly correlated with %LAA_exp_, an index of air-trapping^20^.

In contrast to the emphysema subjects, the dorsal–ventral diaphragm motion decreased in the IPF patients. The decreased lung volume and increased elastic recoil of the lungs would impose mechanical disadvantages not only on the diaphragm but also on the chest wall. It has been shown that the displacement of the chest wall during inspiration is the greatest in dorsal–ventral direction^21^. Thus, decreased chest wall motion would contribute to reduced dorsal–ventral movement in IPF patients. We speculated that this 3D visualization of diaphragmatic motion differences in emphysema and IPF might aid an understanding of the contribution of diaphragm muscles to breathing in patients with chronic respiratory disease.

We measured diaphragm thickness at the posteromedial muscular portion in which diaphragm is located between the lung and peritoneal fat. As a result, diaphragm thickness was smaller in both emphysema and IPF patients than that of normal subjects. These findings are consistent with previous studies by Oancea et al.^22^ and Santana et al.^2^, who demonstrated that patients with COPD or IPF exhibited a decrease in diaphragm thickness or thickening fraction compared with the healthy subjects. In emphysema, pulmonary hyperinflation due to air-trapping results in shortening of contractile fibers, which leads to ineffective contractions^23^. In IPF, variable factors such as a use of steroid, systemic inflammation, hypoxia, and malnutrition might eventually lead to diaphragm atrophy^7,8,24^.

In our study, the mean displacement angle of the whole diaphragm was not different between normal subjects and patient groups. Meanwhile, when analyzed in four quadrants, the posteromedial quadrant in emphysema and the anteromedial quadrant in IPF demonstrated relatively a greater medial displacement compared with the greater ventral displacement in healthy subjects. We speculated that thinning of the posteromedial diaphragm, normally one of the thickest parts near crus, affected the movement angle in emphysema patients. The role of the posteromedial part of the diaphragm might be important for breathing. In contrast, although posteromedial diaphragm thickness was decreased in IPF patients as well, the angle of the anteromedial rather than posteromedial quadrant differed possibly due to the basal lung fibrosis particularly in the postero-basal regions.

The SD of the displacement angle was greater in the patient groups compared to the normal subjects, reflecting disease-induced heterogeneity in directions of diaphragm movement. We assume that the heterogeneous direction of displacement vectors might interfere with the effective movement of the diaphragm. Vice versa, diaphragmatic weakness in the two diseases might contribute to the directional heterogeneity of displacement vectors.

According to previous studies, emphysematous change of lower lung zone significantly correlated with the abnormal motion of diaphragm^11^ and airflow obstruction^25^. In the current study, the heterogeneity of displacement angle was greater, 3D and apico-basal displacements were smaller in diffuse type emphysema than upper lobe predominant type without statistical significance. We suppose that this statistical insignificance is due to the difference in the way of dividing the upper and the lower lobes. While we divided both lungs by anatomical lobes, previous studies divided the whole lung in halves at the carina level or based on volume. In addition, our study population mainly consisted of centrilobular or paraseptal emphysema, which has a predilection for upper lung zone. Therefore, there was only a limited number of lower lobe predominant emphysema. Further studies including a sufficient number of subjects and classifying emphysema distribution in a different way are warranted.

Our study revealed that diaphragmatic movement is associated with pulmonary function. In emphysema patients, 3D and apico-basal diaphragm movement had a tendency to decrease as FEV1/FVC and FEV1, which are the indices to classify severity of COPD^26^, decrease. Interestingly, post-bronchodilator FVC were correlated with diaphragm displacement in IPF patients. Pre-bronchodilator FVC is commonly used measure of IPF disease severity and progression^27,28^. However, according to Deborah et al.^29^, approximately 10% of IPF patients have reversible airflow limitation due to concomitant obstructive lung disease. We assume that post-bronchodilator FVC might be a better index of IPF disease severity, by removing the influence of reversible airflow limitation.

Meanwhile, diaphragm displacement of IPF patients was negatively correlated with the degree of fibrosis, measured either by visual assessment or AMFM-derived texture analysis. Specific fibrosis patterns related to diaphragm movement differed between two measurement methods: GGO in visual assessment, reticular opacities and honeycombing in texture analysis. The reason for this is probably because a large proportion of ground glass opacities were combined with reticulation, making clear distinction between GGO and reticular opacities difficult in visual assessment. In addition, visual interpretation regarding CT pattern of fibrosis is well-known to be susceptible to considerable interobserver variability^30,31^.

There are several limitations of the study. First, due to the retrospective design, various CT scanners were utilized. However, we consider this limitation as insignificant because 89.2% (116/130) of subjects underwent the same CT scanner and all CT images were acquired using multi-detector CT with 1 mm of slice thickness and reconstruction interval. Second, there is a possibility of underestimating diaphragmatic movement if the patient did not fully inhaled or exhaled. Therefore, we excluded patients whose lung volume on expiration was greater than 90% of lung volume on inspiration. Third, only a subset of the IPF patients was pathologically-proven. However, in a routine clinical setting, IPF can be diagnosed without invasive lung biopsy when there are appropriate clinical contexts and typical image patterns on CT. IPF patients who were diagnosed without biopsy in our study showed typical usual interstitial pneumonia (UIP) patterns on CT and restrictive pattern of PFT with insidious onset of dyspnea. Fourth, due to the nature of CT examination, measurements were done in a supine body posture. In an upright posture, diaphragm movement may change due to different diaphragm position and abdominal pressure. Influence of body posture on diaphragm should be taken into consideration in the interpretation of our results. Fifth, different gender composition between patient groups might affect the results of our study. However, to minimize the gender differences, we normalized displacement magnitudes by the cubic root of the global lung volume change from expiration to inspiration CT to cancel out the inter-subject variability. Sixth, this study is based on the assumption that movement of diaphragm is equivalent to movement of the basal lung. It was to avoid validation issues arising from diaphragm segmentation. Instead, we used the automated process of matching lung regions between two CTs, which has been validated and used in a number of previous works^14,15,32–34^.

In conclusion, diaphragm motion is decreased in both emphysema and IPF, but the pattern is different. Apico-basal movements are reduced in emphysema patients while dorsal–ventral movement is reduced in IPF patients. Application of an image registration technique with CT scanning at two lung volumes helps us to understand the pathophysiology of limited diaphragmatic motion in COPD and IPF.

## Methods

Institutional review board of Seoul National University Hospital (No.1806-066-950) approved this retrospective study and the requirement for informed consent was waived. All research was performed in accordance with relevant guidelines and regulations.

### Patients

The patient cohort evaluated in this study was derived from the radiologic database of our institution in Korea. We used the following inclusion criteria: (a) patients who were diagnosed with COPD by PFT as FEV1/FVC (%) < 70% after the use of a bronchodilator; (b) patients who had both inspiratory and expiratory CT scans; (c) %LAA_insp_ (percentage of lung volume at TLC with attenuation less than -950HU) > 10%; (d) a ratio of expiratory air volume to inspiratory air volume less than 0.9, to exclude under-inspiration or under-expiration. We also selected patients with IPF diagnosed by either surgery or clinically with inspiratory and expiratory CT scans. Two chest radiologists (C.L. and K.J.C.) reviewed all CT images to classify them into three categories (UIP, probable UIP, and indeterminate for UIP) according to 2018 American Thoracic Society (ATS) guidelines in consensus^35^. Among them, patients who showed UIP pattern were included in our study. Finally, 50 patients with emphysema (49 men, 1 woman; mean age, 69.5 years; age range, 55–84 years) and 51 patients with IPF (34 men, 17 women; mean age, 70.5 years; age range, 41–87 years) were included in our study. In addition, 29 normal subjects (12 men, 17 women; mean age, 60.9 years; age range, 23–79 years) were included for comparison, who had normal chest CT and PFT results without a past history of pulmonary disease.

### CT Acquisition, image segmentation and registration

All subjects underwent CT scans at full inspiration and full expiration from 16–256 multidetector CT scanners: Ingenuity (n = 116), Brilliance 64 (n = 9), or IQon spectral CT (n = 2); (Philips Medical Systems, Cleveland, OH, USA), Sensation 16 (n = 1), or Somatom Definition Flash (n = 1); (Siemens Medical Solutions, Forchheim, Germany), Revolution CT (n = 1); (General Electric Medical Systems, Milwaukee, WI, USA). The scanning parameters were as follows: rotation time (0.5 s), tube voltage (120 kVp), tube current (170 mAs), slice thickness (1 mm), and reconstruction interval (1 mm).

All volumetric CT images at inspiration and expiration were segmented to extract the airways, vessels, lungs, and lobes using VIDA Apollo 2.0 image processing software (VIDA Diagnostics, Coralville, Iowa, USA). Air and tissue volumes were computed based on the CT density in voxels. %LAA_insp_ and %LAA_exp_ were measured. We defined emphysema distribution index (EDI) of each lung as upper to lower lobe ratio of %LAA_insp_ based on following equations:

$$\mathrm{EDI }\, (\mathrm{left}\,\mathrm{lung})=\frac{\mathrm{\%LAA}_{\text{insp}}\left({\text{LUL}}\right)}{\mathrm{\%LAA}_{\text{insp}}\left(\mathrm{LLL}\right)}$$

$$\mathrm{EDI }\, (\mathrm{right}\,\mathrm{lung})=\frac{\mathrm{\%LAA}_{\text{insp}}\left(\mathrm{RUL}+\mathrm{RML}\right)}{\mathrm{\%LAA}_{\text{insp}}(\mathrm{RLL})}$$

where %LAA_insp_(LUL), %LAA_insp_(LLL), %LAA_insp_(RUL + RML), and %LAA_insp_(RLL) means %LAA_insp_ of left upper lobe (LUL), left lower lobe (LLL), right upper lobe (RUL) plus right middle lobe (RML), and right lower lobe (RLL), respectively. Distribution of emphysema for each side of the lung was divided into two types according to EDI of each lung; upper lobe predominant type (EDI ≥ 2, n = 46), and diffuse type (EDI < 2, n = 54).

**Figure 6:**
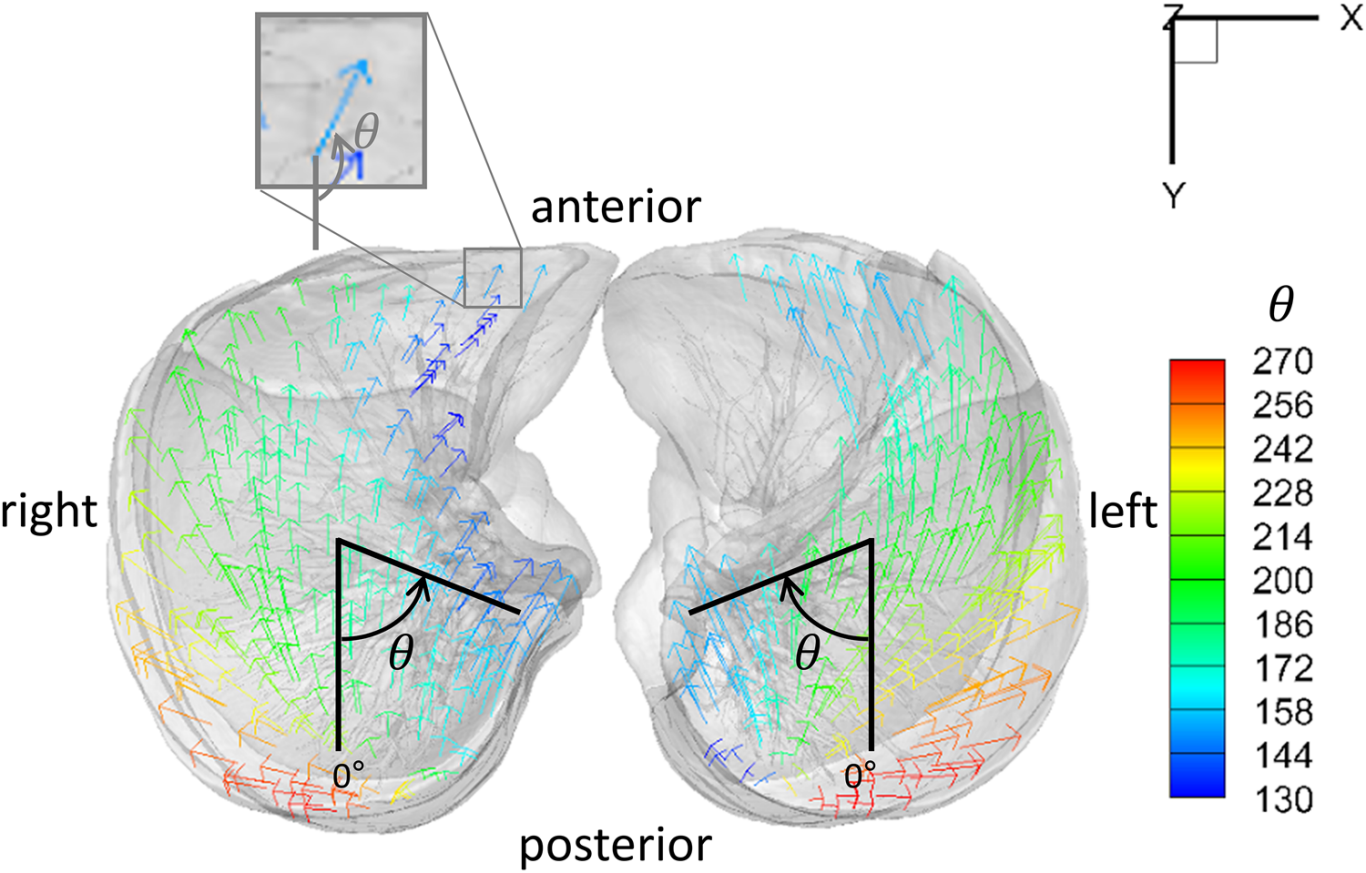
Definition of dorsoventral-transverse displacement angle. Displacement angle was defined by the angle (θ) between dorsoventral-transverse displacement vectors (xy-plane) and dorsal–ventral axis (y-axis), neglecting apico-basal changes. We defined ventral-dorsal direction as zero degrees and the angle is increasing from the inside to the outside. The figure shows the diaphragm viewed from below. Multiple arrows are displacement vectors. The color of the arrows represents displacement angles (θ, Displayed in the color-bar on the right side of the figure).

**Figure 7:**
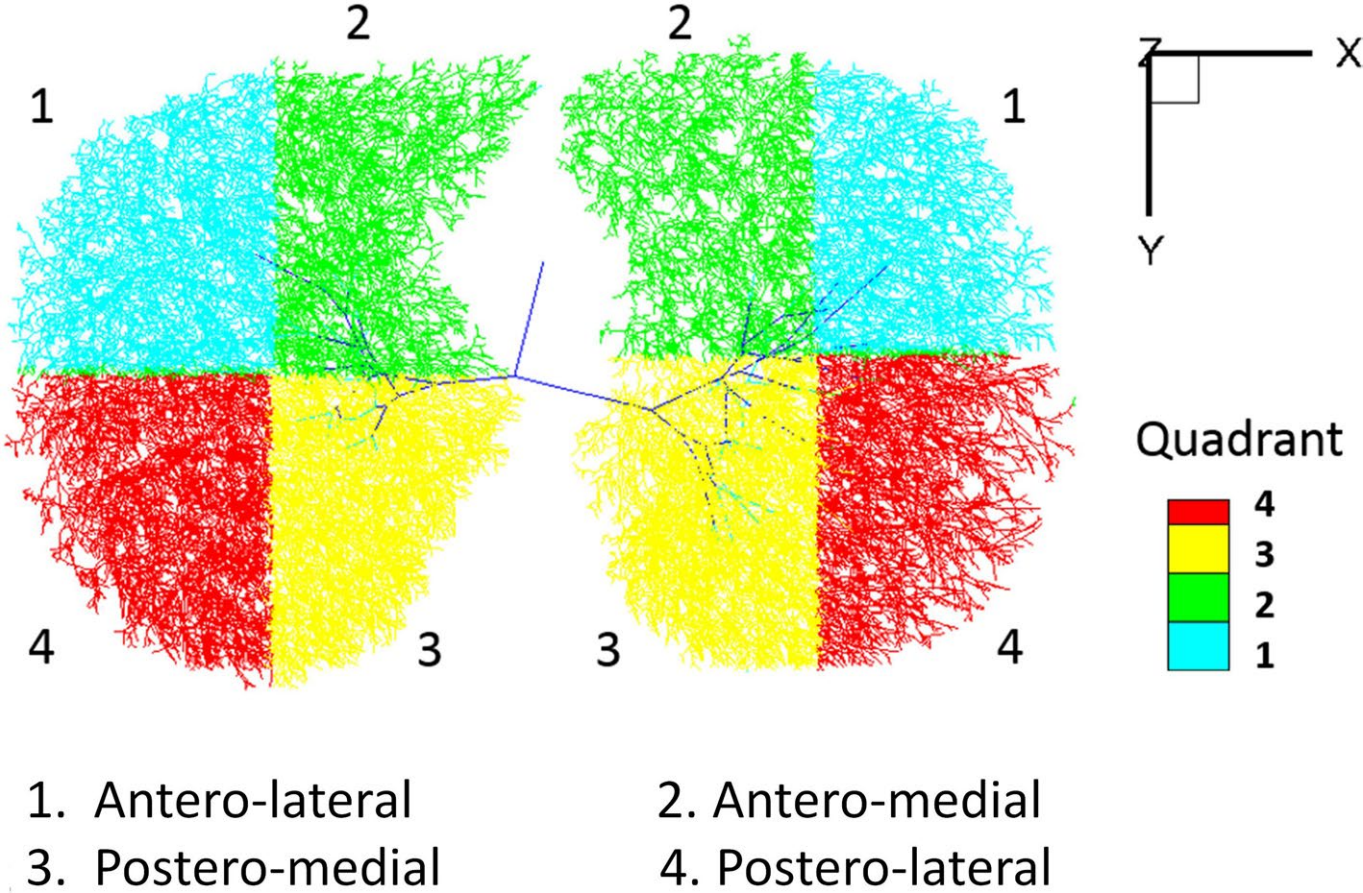
Quadrants of the modeled conducting airways. Quadrants are color-coded by the four divisions of the diaphragms. We defined the center points of the diaphragm on xy-plane by the center points between the maximum and minimum x coordinates and maximum and minimum y coordinates.

Movement of the diaphragm was assumed to be equivalent to movement of the basal lung regions within 5 mm from the diaphragm. Mass preserving non-rigid image registration^14^ was performed to match local lung regions of inspiratory and expiratory CT scans. From matched local lung parenchyma units at an approximate acinar scale based upon the entire conducting airway model^32–34,36–38^, 3D displacement vectors from expiration to inspiration were computed. One-dimensional (1D) displacement magnitudes were also calculated in each of transverse (x-axis), dorsal–ventral (y-axis), and apico-basal directions (z-axis), and 2D components were also computed. Displacement magnitudes were normalized by the cubic root of the global lung volume change from expiration to inspiration CT to cancel out inter-subject variability by inspiration and expiration lung volumes. The normalization formula is as follows:

$${s\left({\varvec{x}}\right)}^{*}=\frac{s\left({\varvec{x}}\right)}{{\left({\mathrm{V}}^{IN}-{\mathrm{V}}^{EX}\right)}^\frac{1}{3}}$$

where s(x)* is the normalized displacement magnitude at local lung region x, s(x) is the displacement magnitude, V^IN^ represents lung volume at inspiration, and V^EX^ is the lung volume at expiration. Furthermore, the directions of displacement vectors were computed by calculating the angle between dorsoventral-transverse displacement vectors (xy-plane) and dorsal–ventral axis (y-axis), neglecting apico-basal changes. We defined the ventral-dorsal direction as zero degrees. The angle increases from the inside to the outside (Fig. 6). The magnitudes and directions of the displacement vectors were computed in each quadrant of both (left and right lung) diaphragms (Fig. 7). The center point of the diaphragm was defined as the center point between the maximum and minimum x coordinates and maximum and minimum y coordinates. An entire conducting airway model^32,36,37^ was utilized to demonstrate the airway trees near the diaphragm.

### Diaphragm thickness

**Figure 8:**
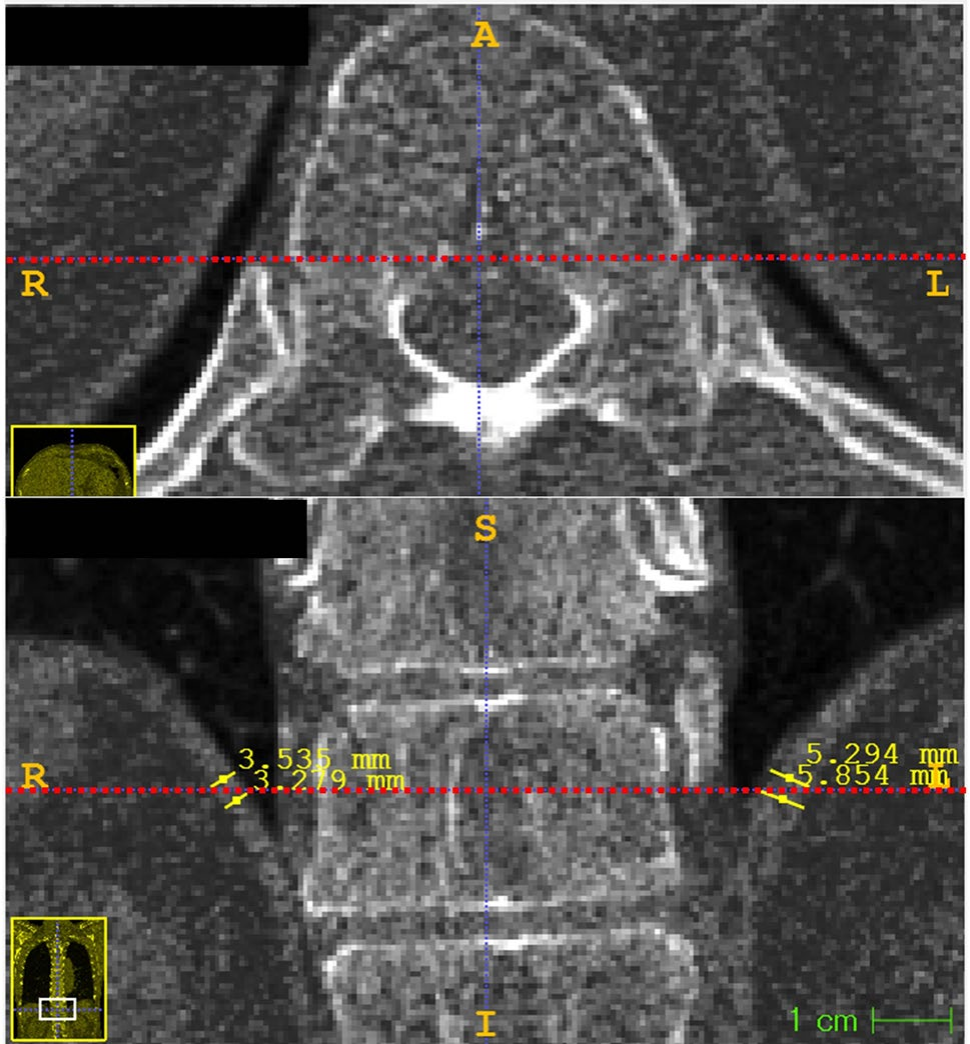
Diaphragm thickness measurement. A line was drawn through the anterior border of the spinal canal at T11 to L1 vertebral body level on the axial image (upper). Software automatically displayed intersection points of the line and diaphragm on the coronal image (lower). Diaphragm thickness was measured at the corresponding points two times on each side of the diaphragm and the mean value was obtained.

One radiologist (J.H.K.) measured diaphragm thickness on inspiratory CT scans using 3D imaging software (ITK-SNAP version 3.6^39^, open-source software). Firstly, a line was drawn through the anterior border of the spinal canal at T11 to L1 vertebral body level. Vertebral body level on which posteromedial diaphragm is distinguishable from adjacent solid organs and shows uniform thickness was selected^40,41^. The software automatically displayed intersection points of the line and diaphragm on coronal images. Diaphragm thickness was measured at corresponding points two times on each side of the diaphragm, and the mean value was obtained (Fig. 8).

### Pulmonary function test

PFTs were performed on the same day as CT scanning in 84 subjects (64.6%, 84/130). Among the other 46 subjects, 30 patients (65.2%, 30/46) underwent pulmonary function test within 30 days from chest CT acquisition. The following variables were obtained: FVC, FEV1, and FEV1/FVC ratio. For 50 emphysema patients (100%, 50/50) and 40 IPF patients (78.4%, 40/51), post-bronchodilator testing was performed with administration of 400 μg of salbutamol by means of inhaler or nebulizer. TLC and residual volume (RV) were measured for 93 subjects (71.5%, 93/130). Diffusing capacity for carbon monoxide (DLCO) was obtained for 104 subjects (80%, 104/130). The results were expressed as the absolute values and the percentage of predicted values.

### Visual and software analysis of IPF

CT images of IPF patients were visually assessed by two radiologists (J.H.K. and C.L.) in consensus. The extents of reticular opacities, honeycombing, and GGO were scored as the percentages of lung parenchyma involved to the nearest 5% in three zones in each lung. The upper zone was defined as above the level of the carina, the middle zone was defined as between the carina and the inferior pulmonary vein confluence, and the lower zone was defined as below the level of inferior pulmonary vein confluence. For each lung, the extent of reticular opacities, honeycombing, and GGO was calculated by averaging the extent obtained at three zones. The sum of all the scores (reticular opacities, honeycombing and GGO) in each lung was defined as a total score.

In addition, we performed lung texture analysis with the use of the AMFM software^42–45^. AMFM software measured the proportions of lung volume occupied by reticular opacities, honeycombing, and GGO.

### Statistical analysis

The differences in displacement vectors and diaphragm thickness between normal subjects and emphysema patients, normal subjects and IPF patients, emphysema and COPD patients were evaluated with the independent t-test. ANOVA was performed to determine whether significant differences existed among the three groups. For post hoc analysis, the holm test was used when the ANOVA test was statistically significant. For emphysema patients, upper lobe predominant and diffuse types were compared using independent t-test. Pearson’s correlation test was used to correlate %LAA_insp_, %LAA_exp,_ PFT data, score of IPF (reticular opacities, honeycombing, and GGO), with the magnitude and the angle of displacement vectors. Statistical analyses were performed with a free software package (R statistical programming environment, version 3.0.2; the R foundation, Vienna, Austria). A two-tailed *P* value less than 0.05 was considered to indicate a statistical significance.

## Data Availability

The datasets used and analysed during the current study are available from the corresponding author on reasonable request.
